# Autistic traits in psychotic disorders: prevalence, familial risk, and impact on social functioning

**DOI:** 10.1017/S0033291720000458

**Published:** 2021-07

**Authors:** Tim B. Ziermans, Frederike Schirmbeck, Floor Oosterwijk, Hilde M. Geurts, Lieuwe de Haan, Genetic Risk and Outcome of Psychosis (GROUP) Investigators

**Affiliations:** 1 Department of Psychiatry, Amsterdam UMC, University of Amsterdam, Amsterdam, The Netherlands; 2 Department of Psychology, University of Amsterdam, Amsterdam, The Netherlands; 3 Arkin Institute for Mental Health, Amsterdam, The Netherlands; 4 Parnassia, Zaandam, The Netherlands; 5 Dr. Leo Kannerhuis, Amsterdam, The Netherlands

**Keywords:** autism, functional outcome, mentalizing, psychosis, schizophrenia, social cognition

## Abstract

**Background:**

Prevalence estimates of autistic traits in individuals with psychotic disorders (PD) vary greatly and it is unclear whether individuals with a familial risk (FR) for psychosis have an increased propensity to display autistic traits. Furthermore, it is unknown whether the presence of comorbid autism traits disproportionally affects the cognitive and behavioral aspects of social functioning in PD.

**Methods:**

In total, 504 individuals with PD, 587 unaffected siblings with FR, and 337 typical comparison (TC) individuals (16–50 years) were included. Autistic and psychotic traits were measured with the Autism Spectrum Quotient (AQ) and the Community Assessment of Psychic Experiences (CAPE). Social cognition was assessed with the Picture Sequencing Task (PST) and social behavior with the Social Functioning Scale (SFS).

**Results:**

For PD 6.5% scored above AQ clinical cut-off (⩾32), 1.0% for FR, and 1.2% for TC. After accounting for age, sex, and IQ, the PD group showed significantly more autistic traits and alterations in social behavior and cognition, while FR and TC only displayed marginal differences. Within the PD group autistic traits were a robust predictor of social behavior and there were no interactions with positive psychotic symptoms.

**Conclusions:**

Levels of autistic traits are substantially elevated in PD and have a profoundly negative association with social functioning. In contrast, autistic traits above the clinical cut-off are not elevated in those with FR, and only marginally on a dimensional level. These findings warrant specific clinical guidelines for psychotic patients who present themselves with autistic comorbidity to help address their social needs.

## Introduction

Since the concept of ‘autism’ was conceived by Bleuler in 1911 (Bleuler, 1950), it has been intertwined with the diagnosis of schizophrenia and evolved from a central feature of severe psychotic disorders (PD) to a separate spectrum of clinical disorders. The literature suggests that autism spectrum disorders (ASD) and PD still co-occur more frequently than one would expect based on the prevalence rates in the general population, which gravitate around 1% for both (Chisholm, Lin, Abu-Akel, & Wood, 2015). In addition, findings from longitudinal, registry-based studies convincingly suggest that individuals diagnosed with ASD are not only at an increased risk for non-affective PD such as schizophrenia, but also for affective PD, as observed in diagnosed mood disorders (Schalbroeck, Termorshuizen, Visser, Van Amelsvoort, & Selten, 2018; Selten, Lundberg, Rai, & Magnusson, 2015). *Vice versa*, the prevalence of ASD diagnoses in individuals with an established PD has been investigated less systematically and is mostly restricted to the observations in adult populations of modest sample size (Chisholm et al., 2015; Kincaid, Doris, Shannon, & Mulholland, 2017; Padgett, Miltsiou, & Tiffin, 2010). Findings from the largest patient sample to date indicate a substantially elevated prevalence (3.6%, *N* = 197) (Davidson, Greenwood, Stansfield, & Wright, 2014) of ASD diagnoses among PD individuals, but this analysis did not include the prevalence estimates of sub-clinical autism traits.

Recent efforts have therefore focused more on dimensional rather than categorical approaches, under the assumption that both conditions represent extremes on a continuum of symptomatic severity, and that even isolated or low-intensity traits may affect clinical outcomes. Such studies have provided further evidence that autistic and psychotic traits co-occur at an elevated behavioral level in clinical samples (Barneveld et al., 2011; De Crescenzo et al., 2019; Esterberg, Trotman, Brasfield, Compton, & Walker, 2008; Fagel et al., 2013; Kincaid et al., 2017; Ziermans, Swaab, Stockmann, de Bruin, & van Rijn, 2017). From a cognitive and functional perspective, it is also well established that both ASD and PD are characterized by (partially) overlapping impairments compared to typical healthy comparisons, particularly in the social domain (Martinez et al., 2017; Pinkham et al., 2019; Sasson et al., 2007, 2011, 2016; Velthorst et al., 2018). However, it is currently unclear whether the presence of comorbid autistic and psychotic traits has a detrimental effect on the outcome. A recent study in schizophrenia patients with a positive screening for ASD (28 out of 75) showed an increased duration of illness, more general psychopathology, and worse cognitive functioning (working memory and processing speed) compared to patients without ASD traits, yet similar levels of IQ, social cognition, and functioning (Barlati, Deste, Gregorelli, & Vita, 2019). In contrast, Vaskinn and Abu-Akel (2019) used a dimensional approach and concluded that the presence of more psychotic and autistic traits in schizophrenia patients (*N* = 81) was associated with relatively better functioning and mentalizing. The latter finding also provided clinical support for the (counterintuitive) diametrical model of autism and psychosis (Crespi & Badcock, 2008), which posits that both disorders have opposite genetic relations and that co-occurrence would diametrically modulate behavior toward normality.

Whether genetic markers associated with autism and psychosis are diametrical opposites remains open to debate. There is ample evidence, however, that etiological overlap between both conditions does exist, as further supported by recent studies focusing on polygenic risk for both autism and schizophrenia (Fromer et al., 2016; Pourcain et al., 2018; Velthorst et al., 2018), as well as the presence of rare copy-number variations in both populations (Kushima et al., 2018; Zarrei et al., 2019). In addition, given the highly heritable nature of both ASD and PD, it is reasonable to expect a higher co-occurrence of subclinical traits of both dimensions in unaffected relatives of either patient population. Indeed, twin data have suggested that psychiatric disorders in general are associated with continuously distributed genetic risks throughout the general population (Taylor et al., 2019). An important next step is to establish whether concurrent traits of autism and psychosis are also associated with social impairments in at-risk samples.

The primary aim of the current study was to establish the prevalence of autistic traits in a substantial group of PD individuals compared to their unaffected siblings with a familial risk for PD (FR) and a typical comparison (TC) group. It was expected that the PD group would show more autistic traits than the TC group, with a large effect size, and the FR group in an intermediate position. The second aim was to establish to what extent the levels of autism or psychotic traits were associated with social cognition and social functioning, using both categorical and dimensional approaches. It was expected that both psychotic and autism traits would show independent negative associations with social cognition and functioning across groups, but most profoundly within the PD group as we assumed they would display more variability in trait scores and therefore stronger associations. A third and final aim was to explore whether our data could provide any further support for the diametric hypothesis, which postulates that negative main effects of both autistic and positive psychotic traits on social functioning are modulated toward positivity if both are present.

## Methods

The current cross-sectional study is part of a longitudinal cohort study, Genetic Risk and Outcome of Psychosis (GROUP). The main objective of GROUP is to elucidate etiological and pathogenetic factors influencing the onset and course of PD in patients, their unaffected family members, and non-related controls. For a full description of recruitment and assessment procedures, the reader is referred to a previous publication on this topic (Korver et al., 2012).

### Participants

Participant groups for this study consisted of patients diagnosed with schizophrenia or related PD, unaffected siblings with FR and TC individuals from the general population. Participants were recruited in 36 mental health care institutes in the Netherlands and Belgium. PD were identified through clinicians in the participating institutes by applying the following inclusion criteria: (1) age between 16 and 50 years, (2) meet DSM-IV-TR (American Psychiatric Association, 2000) criteria for a non-affective PD, (3) good command of the Dutch language, and (4) able and willing to provide informed consent. Siblings were not allowed to meet the criteria for a lifetime diagnosis of any PD at baseline. Healthy control participants had no lifetime diagnosis of a PD at baseline and no first-degree relative with a lifetime PD. For the purpose of the present study, we only included participants from the database (Data release 6.0) with available data on the Autism Spectrum Quotient (AQ) (Baron-Cohen, Wheelwright, Skinner, Martin, & Clubley, 2001) measured at the 6-year follow-up assessment (T3). The study was approved by the Medical Ethics Committee of the University Medical Center Utrecht and subsequently by local review boards of each participating institute. All subjects gave written informed consent in accordance with the committee's guidelines.

### Instruments

#### Global cognitive functioning

All participants were assessed with a cognitive task battery that included four tasks (Arithmetic, Block Design, Digit Symbol-Coding, and Information) of the Dutch version of the Wechsler Adult Intelligence Scale – Third Edition (WAIS-III; Wechsler, 1997), resulting in a total IQ estimate.

#### Psychotic traits

Psychotic traits were assessed with the Community Assessment of Psychic Experiences (CAPE; Stefanis et al., 2002). This self-report scale typically measures the lifetime prevalence of positive, negative, and depressive traits on both a frequency scale and a distress scale. Because the instrument was assessed as part of a follow-up study, individuals were instructed to answer the questions with regard to the last 3 years (since the previous assessment). Only the more commonly used frequency scores were included in this study.

#### Autistic traits

The AQ is a well-validated, 50-item self-report questionnaire that measures autism traits and is commonly used to screen for ASD diagnosis (Woodbury-Smith, Robinson, Wheelwright, & Baron-Cohen, 2005). AQ scores fall within the range of 0–50 and higher scores indicate a higher level of autism traits. In addition, AQ scores of ⩾32 indicate that an ASD diagnosis may be appropriate, and individuals with ASD are unlikely to score below a threshold of 26 (Baron-Cohen et al., 2001; Woodbury-Smith et al., 2005). The AQ consists of five subscales measuring the following domains: social skills, communication, imagination, attention switching, and attention to detail. The Dutch translation of the AQ was used for this study (Hoekstra, Bartels, Cath, & Boomsma, 2008).

#### Social functioning

*Cognition.* The Picture Sequencing Task (PST; Langdon and Coltheart, 1999) was assessed to measure mentalizing ability in participants. The PST consists of stories depicted in four illustrated (black and white) card picture sequences. Subjects are asked to turn over the cards placed in front of them and to place them in the correct order to show a logical sequence of events. The false-belief (PST-FB) stories depict a story character who, unaware of an event that occurred in a story, acted on his or her misinformation. These stories have previously been associated with underperformance in individuals with schizophrenia (Langdon, Coltheart, & Ward, 2006) and autism (Baron-Cohen, Leslie, & Frith, 1986). Total score ranges between 0 and 24.

*Behavior.* On a daily behavioral level, social functioning was assessed with the Social Functioning Scale (SFS; Birchwood, Smith, Cochrane, Wetton, and Copestake, 1990), a widely used self-report instrument to measure the areas of functioning essential for successful community maintenance. The total scaled score on the SFS was used as a measure of overall social functioning in the past 3 months. Higher scores on the SFS indicate higher levels of social functioning. It has a mean standardized score of 100, with a standard deviation of 15. It is commonly assessed in PD, and adults with ASD have an equally reduced score on this instrument compared to TC (Chan et al., 2019).

### Statistical analysis

The χ^2^ and ANOVA tests were used for the comparisons of group characteristics, with Cramer's *V* and partial *η*^2^ as respective effect sizes, followed by Games–Howell or Hochberg's G2 *post hoc* tests, where applicable. To compare the prevalence of autism and psychotic traits (AQ and CAPE) across groups, a missing value analysis was conducted and missing items were imputed with the predicted mean matching method (Markov chain Monte Carlo; 50 iterations) to generate subscale and total scores for all individuals. Proportions above cut-off scores were compared with χ^2^ tests. Next, (M)ANCOVA was implemented by using general linear models with age, sex, and IQ entered as covariates and Bonferroni-corrected *post hoc* comparisons.

Next, we assessed the relative impact of autism traits on social functioning across groups by conducting two separate series of multilevel mixed linear models for both dependent variables. A two-level model with random intercepts was applied, i.e. measurements within families and within study sites. Age, IQ, and trait scores were centered around their respective means. In the initial models, group was entered as a factor. Significant analyses were continued for PD only, as this was the primary group of interest. The relative goodness of fit of the competing models was evaluated with the Akaike information criterion (AIC) and the Bayesian information criterion (BIC), where smaller values indicate a better fit. Finally, to test for a possible diametric effect between positive symptoms and autistic symptoms, the analyses were repeated with AQ Total, CAPE positive, and their interaction term included. Multilevel models were computed with the unstructured covariance setting and maximum likelihood estimation.

To improve reproducibility and reduce the chance of Type I error, a critical *p* value of <0.005 was applied for all *ad hoc* analyses (Benjamin et al., 2018) and *p* < 0.05 for *post hoc* and explorative analyses. Cohen's *d*, based on pooled variances and estimated marginal means, was calculated for the effect sizes of group differences and the standardized *β* coefficient was reported as an effect size for individual predictors in multilevel analyses.

## Results

### Demographics and clinical characteristics

Data were available for 504 PD, 572 FR, and 337 TC individuals (*N* = 1413). Demographic data and clinical characteristics are provided in Table 1. Groups differed significantly on sex, age, education, and IQ estimate (all *p* < 0.001). *Post hoc* testing showed that there were significantly more males in the PD group than in the FR (χ^2^ = 161.16, *p* < 0.001, *V* = 0.28) or TC group (χ^2^ = 129.89, *p* < 0.001, *V* = 0.30). Both the PD and FR groups had a lower mean age than the TC group (both *p* < 0.001, *η^2^*_partial_ = 0.08 and 0.06) and education (*post hoc*, all *p* < 0.001, *V*_range_ = 0.14–0.44) and IQ estimates differed for all group comparisons (*post hoc*, all *p* < 0.01, *η^2^*_partial, range_ = 0.01–0.14) with PD showing the lowest average IQ score and TC the highest.

**Table 1.** Demographic and clinical characteristics of patients, siblings, and healthy controls in the GROUP study, mean scores (standard deviations) and absolute numbers (%)

	PD (504)	FR (572)	TC (337)	Statistic	*p* value	ES	*Post hoc*
Sex (male)	365 (72.4)	254 (44.4)	153 (42.4)	χ^2^ = 108.17	<0.001	*V* = 0.27	PD > FR & TC
Age (years)	33.4 (7.2)	34.0 (7.9)	38.5 (10.6)	*F* = 30.89	<0.001	*η^2^_p_* = 0.06	PD & FR < TC
Age of onset (years)^b^	22.4 (6.5)	–	–				
Illness duration (years)	11.5 (4.3)	–	–				
Estimated IQ^b^	100.7 (17.9)	111.5 (17.7)	115.4 (17.1)	*F* = 83.15	<0.001	*η^2^_p_* = 0.11	PD < FR < TC
Education				χ^2^ = 198.45	<0.001	*V* = 0.26	PD < FR < TC
Low	133 (26.4)	52 (9.1)	11 (3.3)				
Middle	260 (51.6)	227 (39.7)	116 (34.4)				
High	111 (22.0)	293 (51.2)	210 (62.3)				
CAPE scores^a^^,^^b^							
Positive	29.3 (9.7)	21.7 (2.5)	21.5 (3.0)	*F* = 493.83	<0.001	*η^2^_p_* = 0.26	PD > FR & TC
Negative	26.2 (7.8)	20.7 (5.7)	19.7 (4.8)	*F* = 280.57	<0.001	*η^2^_p_* = 0.17	PD > FR > TC
Depression	15.1 (4.8)	12.1 (3.3)	11.8 (3.3)	*F* = 203.81	<0.001	*η^2^_p_* = 0.13	PD > FR & TC
DSM-IV Diagnosis^c^							
Schizophrenia	310 (61.5)	–	–				
Schizophreniform d.	36 (7.1)	–	–				
Schizoaffective d.	67 (13.3)	–	–				
Psychotic d.	79 (15.7)	–	–				
Delusional d.	12 (2.4)	–	–				
In remission >6 months^b^^,^^d^							
Yes/no/unknown	198/274/29						
Antipsychotic medication							
Yes/no/unknown	345/4/155	–	–				

PD, psychotic disorder group; FR, familial risk group; TC, typical comparison group; ES, effect size.

a Games–Howell, *p* < 0.05.

b Missing data: Age of onset – 1 PD, IQ – 1 FR, CAPE – 2 PD & 3 FR, PANSS – max. 20 PD.

c Based on the Comprehensive Assessment of Symptoms and History (CASH; Andreasen, Flaum, and Arndt, 1992) and the Schedules for Clinical Assessment for Neuropsychiatry (SCAN 2.1; Wing et al., 1990) at baseline, reported for schizophrenia spectrum only.

d Based on PANSS remission tool (Andreasen et al., 2005).

In the PD group, schizophrenia diagnoses were the most common primary DSM-IV diagnosis (61.5%), followed by psychotic (15.7%) and schizoaffective (13.7%) diagnoses. Psychotic traits on all three CAPE dimensions (Positive, Negative, and Depressive) were significantly more frequent in PD compared to FR and TC (all *p* < 0.001, *η^2^*_partial, range_ = 0.12–0.23). In addition, FR reported significantly more negative traits than TC on average (*p* = 0.018, *η^2^*_partial_ = 0.01), but no difference in positive (*p* = 0.561, *η^2^*_partial_ = 0.00) or depressive traits (*p* = 0.542, *η^2^*_partial_ = 0.00).

### Autistic traits

**Fig. 1.**
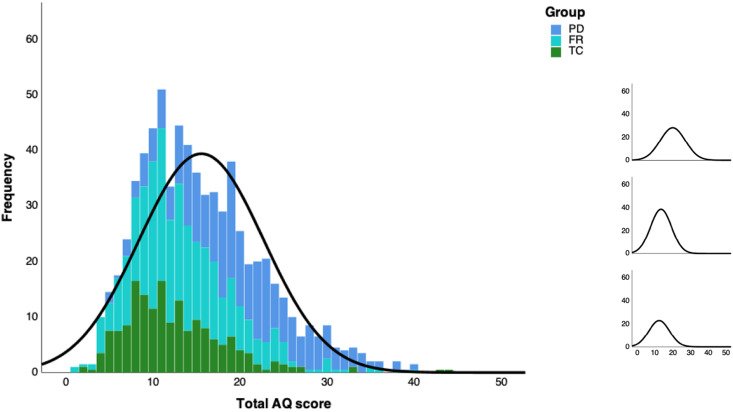
Left: Histogram plot with the normal curve of Total AQ scores stacked across groups. Right: Normal curves per group: PD (top), FR (middle), and TC (bottom).

Missing value analysis indicated that for 54 individuals (3.8%; 31 PD, 17 FR, 6 TC) AQ data were incomplete, with a total of 0.1% of items missing. Due to the small proportion of missing values, a single imputed data set was generated. Total AQ score distributions are illustrated in Fig. 1 and reported by group and gender for all subscales in Table 2. Based on recommended and most stringent clinical cut-offs for Total AQ scores (Table 2), 6.5% of PD scored ⩾32 and 21.4% ⩾26, while lower proportions scored above these thresholds in the FR (1.0% and 2.8%) and TC (1.2% and 2.3%) groups, respectively. Proportions differed significantly across all groups for AQ ⩾ 32 (χ^2^ = 32.62, *p* < 0.001, *V* = 0.15) and AQ ⩾ 26 (χ^2^ = 135.19, *p* < 0.001, *V* = 0.31). *Post hoc* comparisons showed that PD differed from the other groups on both cut-offs (all *p* < 0.001, *V*_range_ = 0.13–0.27), but FR did not differ from TC (*p* = 0.85, *V* = 0.01 and *p* = 0.70, *V* = 0.01, respectively).

**Table 2.** AQ data per group, mean scores (standard deviations) and number of participants exceeding standard AQ cut-off scores (%)

		PD			FR			TC	
	Total (504)	Male (365)	Female (139)	Total (572)	Male (254)	Female (318)	Total (337)	Male (143)	Female (194)
AQ Total (0–50)	20.04 (7.06)	20.18 (6.81)	19.68 (7.69)	13.48 (5.89)	14.42 (6.39)	12.72 (5.36)	12.43 (5.82)	13.50 (5.76)	11.64 (5.75)
Social skill	3.57 (2.35)	3.48 (2.31)	3.80 (2.44)	1.98 (1.96)	2.06 (2.15)	1.91 (1.79)	1.75 (1.82)	1.81 (1.92)	1.70 (1.75)
Communication	3.27 (1.96)	3.28 (1.94)	3.23 (2.02)	1.97 (1.69)	2.19 (1.87)	1.80 (1.52)	1.82 (1.64)	2.22 (1.66)	1.53 (1.57)
Imagination	4.19 (2.00)	4.45 (1.84)	3.51 (2.23)	3.11 (1.80)	3.55 (1.81)	2.76 (1.72)	2.93 (1.77)	3.21 (1.88)	2.72 (1.66)
Attention to detail	4.03 (2.20)	4.00 (2.23)	4.11 (2.10)	3.33 (1.87)	3.34 (1.91)	3.32 (1.84)	3.15 (1.93)	3.14 (1.99)	3.15 (1.88)
Attention switching	4.99 (2.35)	4.06 (2.31)	5.03 (2.22)	3.08 (1.97)	3.28 (2.02)	2.93 (1.92)	2.79 (2.01)	3.12 (2.14)	2.54 (1.87)
AQ ⩾ 32 (%)	33 (6.55)	24 (6.58)	9 (6.47)	6 (1.05)	5 (1.97)	1 (0.31)	4 (1.18)	2 (1.40)	2 (1.03)
AQ ⩾ 26 (%)	108 (21.43)	80 (21.92)	28 (20.14)	16 (2.80)	10 (3.94)	6 (1.89)	8 (2.36)	4 (2.80)	4 (2.06)

Next, mean comparisons showed that groups differed significantly on the overall level of autistic traits, covaried for age, sex, and IQ (*F*_2,1406_ = 129.28, *p* < 0.001, *η^2^*_partial_ = 0.16). Covariates were significant as well, with positive effects for age (*p* = 0.004, *η^2^*_partial_ = 0.01) and male sex (*p* < 0.001, *η^2^*_partial_ = 0.01), and a negative effect for IQ (*p* < 0.001, *η^2^*_partial_ = 0.01). Bonferroni-corrected *post hoc* comparisons indicated that PD reported significantly more autistic traits than FR (*p* < 0.001, *d* = 0.80) and TC (*p* < 0.001, *d* = 0.92). FR also reported more autistic traits than TC, albeit with a small effect size (*p* = 0.028, *d* = 0.18). On a subscale level MANCOVA analysis showed similar group differences on all subscales (all *p* < 0.001), except *post hoc* tests were not significant for mean differences between FR and TC.

### Impact of autistic traits on social functioning in PD

Group, Total AQ, and CAPE Positive were entered by default in the initial models. Linearity assumptions (*p* < 0.05; correlations in online Supplementary Tables) were checked next to determine which additional variables were entered into the models. In the initial models only main effects were included. Additional two-way interactions were also examined for potential model optimization.

**Fig. 2.**
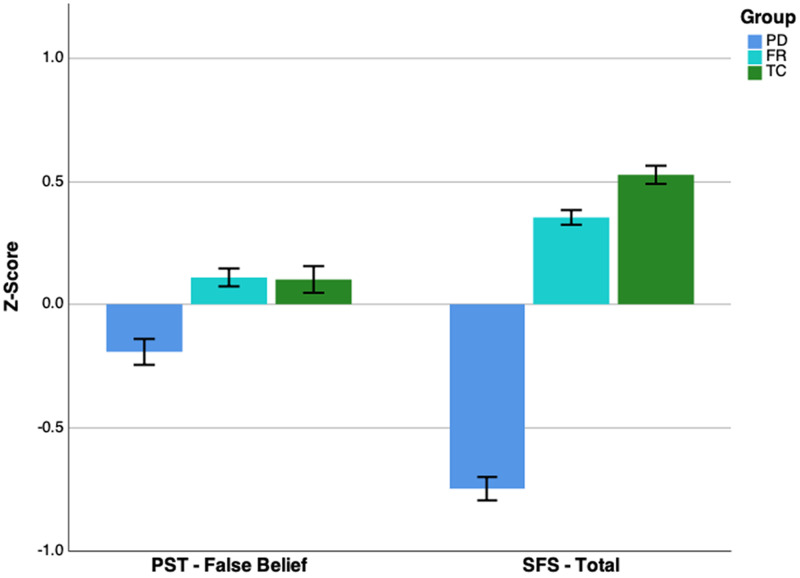
*Z*-transformed group means for Picture Sequencing Test – False Belief (PST-FB; left) and the Social Functioning Scale (SFS; right). Error bars represent ±1 standard error.

*Cognition.* PST-FB data were missing for 55 individuals (17 PD, 18 FR, 20 TC). Standardized group means are displayed in Fig. 2. In addition to Group, Total AQ, and CAPE Positive, the covariates age and IQ were also included. There was no significant group effect on PST-FB (*p* = 0.124), hence model comparison was continued for the total sample without Group as a factor. The best fit (see Table 3*a*) included: Total AQ (*β* = −0.09, *p* = 0.001), age (*β* = −0.20, *p* < 0.001), IQ (*β* = 0.28, *p* < 0.001), and Total AQ × age (*β* = −0.07, *p* = 0.002). CAPE Positive was not significant and no longer included in the final model.

**Table 3.** Best-fitting multilevel models for social functioning

	*B*	s.e.	*p*	95% CI
(*a*) Dependent: picture sequencing task – false belief stories (total sample)				
Intercept	19.676	0.116	<0.001	19.450–19.903
Total AQ	−0.051	0.015	0.001	−0.080 to −0.021
Age	−0.010	0.013	<0.001	−0.125 to −0.021
IQ	0.064	0.006	<0.001	0.052–0.076
Total AQ × age	−0.006	0.002	0.002	−0.009 to −0.002
Centered around the means: Total AQ = 15.57, age = 34.85, IQ = 108.59.				

	*B*	s.e.	*p*	95% CI
(*b*) Dependent: social functioning scale – total score (PD sample only)				
Intercept	112.772	0.376	<0.001	112.034–113.510
Total AQ	−0.473	0.048	<0.001	−0.568 to −0.378
Age	−0.106	0.045	0.019	−0.194 to −0.018
Sex	4.104	0.707	<0.001	2.716–5.493
IQ	0.093	0.018	<0.001	0.057–0.128
CAPE Negative	−0.283	0.002	<0.001	−0.368 to −0.199

Centered around the means: Total AQ = 20.04, age = 33.38, IQ = 100.72, CAPE Negative = 26.25.

*Behavior.* SFS data were missing for 10 individuals (four PD, four FR, two TC). See Fig. 2 for standardized group means. All candidate predictors were included. There was a significant group effect on SFS (*F* = 45.70, *p* < 0.001). Bonferroni-corrected *post hoc* comparisons indicated that PD differed significantly from the other groups (*p* < 0.001), but FR did not differ from TC. Model comparison was then continued for the PD group only. The best model fit (see Table 3*b*) included fixed effects for age (*β* = −0.09, *p* = 0.019), sex (*β* = 0.21, *p* < 0.001), IQ (*β* = 0.19, *p* < 0.001), Total AQ (*β* = −0.37, *p* < 0.001), and CAPE Negative (*β* = −0.24, *p* < 0.001). Neither CAPE Positive and CAPE Depressive, nor any two-way interactions improved both AIC and BIC values.

*Interaction autistic and positive symptoms*. Adding the interaction term Total AQ × CAPE Positive did not yield significant predictors or model improvements for PST-FB or SFS. To allow for better statistical comparison with previous results from Vaskinn and Abu-Akel (2019), the analyses were repeated in generalized linear models for PD only, without region and family ID included in the models. Again, no significant interactions supporting a diametrical model were detected (see online Supplementary Tables).

## Discussion

The current study provides robust evidence for a substantially increased presence of autistic traits in individuals diagnosed with PD compared to their unaffected siblings and TC individuals. This concerns both categorical and dimensional levels of autistic traits, which may arguably reflect separate etiologies (Abu-Akel, Allison, Baron-Cohen, & Heinke, 2019; Linscott & van Os, 2010). Concerning categorical classifications, an AQ cut-off of 32 is known to discriminate satisfactorily between ASD and TC individuals, and a cut-off of 26 shows reasonable sensitivity (Baron-Cohen et al., 2001; Woodbury-Smith et al., 2005). Therefore, our findings suggest that one in 15 individuals with PD falls above the clinical cut-off, and one in five above the sub-clinical threshold. For FR, the odds are, respectively, 6 and 7 times smaller than for PD, and comparable with TC. However, the predictive diagnostic accuracy of AQ thresholds in suspected ASD appears modest (Ashwood et al., 2016), and in our sample, only six (1.2%) PD individuals had a suspected diagnosis of ASD at intake. This raises the question whether elevated autistic traits in PD may reflect symptomatic overlap, in particular with negative psychotic traits. Based on the assessments used in this study, there is evident overlap between the social skill subscale of AQ (10 items; e.g. ‘*I enjoy meeting new people*’) and social withdrawal items of the CAPE (four items; e.g. ‘*Do you ever feel you have no interest to be with other people*’). Nevertheless, most items do not resemble each other as closely and correlations of AQ and CAPE in PD individuals were modest and comparable for all dimensions (0.32–0.36; online Supplementary Table). Therefore, symptom overlap can only provide a limited explanation for the increased presence of autistic traits in PD. Regardless, the absence of diagnoses in our sample further emphasizes that comorbid ASD is potentially overlooked or wrongfully dismissed by clinicians presented with PD cases.

On a dimensional level, group differences in autism traits are equally striking for PD and in line with previous findings in smaller samples (Lugnegard, Hallerbäck, & Gillberg, 2015; Sasamoto et al., 2011). Additionally, individuals with FR reported marginally more autism traits than TC when the potential effects of age, sex, and IQ were accounted for. However, this group difference dissipated on a subscale level. Importantly, our sibling group was unaffected in terms of schizophrenia spectrum diagnoses, but not completely devoid of other comorbid conditions, in particular mood disorders (*n* = 111, including 74 remissions). Explorative *post hoc* analyses indicated that those who met the criteria for any DSM-IV disorder (*n* = 126; *M* = 15.48) scored significantly higher on AQ than those not meeting any criteria (*n* = 446, *M* = 12.91, *p* < 0.001). In addition, the few siblings who did acquire a PD after baseline were reassigned to the patient group. This likely led to a slight underestimation of psychotic and autistic traits in the sibling group than would be expected based on family history alone (Sullivan et al., 2012). As such, we can ascertain that only unaffected siblings of individuals with PD are relatively resistant to developing autistic traits.

As expected, the results provide strong evidence for a profoundly negative relation of autism traits with social functioning in PD. Comparable data are surprisingly scarce or only available for modest and mostly pediatric samples, with three notable exceptions that all used the PANSS Autism Severity Score (PAUSS; Kästner et al., 2015) to measure autism symptoms in adult PD. Barlati et al. (2019) showed that psychosocial functioning did not differ between schizophrenia patients (*N* = 75) with and without autism, after patients were thoroughly screened for ASD. However, global measures of functioning were used (Honos, GAF) and the prediction of autism severity scales was dichotomized, making it difficult to establish any potential linear dosage effects on a dimensional scale. Vaskinn and Abu-Akel (2019) did report a negative linear relation between autistic symptoms and functioning in PD (*N* = 81) by using both GAF and SFS as outcome measures, but also found positive two-way interaction effects with positive symptoms that were suggestive of diametric effects on functioning (discussed further below). Finally, Harvey et al. also found a small but significant effect of autistic symptoms on real-world functioning in PD (*N* = 171) (Harvey et al., 2019). Our results add to these findings a more robust outcome and direct comparisons with a related and unrelated group of individuals without PD.

Although it is known that cognitive and negative symptoms are better predictors of functional outcome (Malla & Payne, 2005; Stouten, Veling, Laan, van der Helm, & van der Gaag, 2017), it was still somewhat unexpected that there were no significant relations between positive psychotic traits and social functioning. The positive traits were also relatively low in our PD sample, perhaps due to treatment effects, which may help explain the absence of associations with functioning measures. To improve our understanding of the dynamics between co-occurring autistic traits, psychotic traits, and functioning in PD, it would therefore be of great interest to study the strength of relations on a subdomain level and determine centrality measures within a network approach (van Rooijen et al., 2018; Wigman et al., 2015; Wüsten et al., 2018).

Social cognitive functioning was also negatively associated with autistic traits in line with previous findings (Harvey et al., 2019; Vaskinn & Abu-Akel, 2019) but, contrary to expectations, the effect was small and comparable across groups. Likewise, the correlation between social cognition and social functioning was surprisingly small. We used a classic mentalizing (false belief) paradigm, which is typically performed less accurately by individuals with PD (Langdon et al., 2006), though not always (Pepper et al., 2018). Group differences in mentalizing were obscured by relatively strong effects of IQ and age in our study. However, these differences may be meaningful in their own right and covarying for them may not always be necessary or preferable in some instances (Miller & Chapman, 2001). The influence of other factors affecting outcome may also help explain why mentalizing performance may not be sensitive enough to reveal strong relations with the functional outcome (Ohmuro et al., 2016). Notwithstanding, it is safe to conclude from our results that autistic traits are negatively associated with mentalizing abilities in general, hence also for PD. Possibly, the effect would have been stronger if other, lower-order social cognition tasks (i.e. emotion recognition) were used, as these appear to be more clearly impaired in both PD and ASD (Eack et al., 2013; Pepper et al., 2018; Sasson, Pinkham, Weittenhiller, Faso, & Simpson, 2016). Future studies may benefit in general from broader and more ecologically valid assessments of social cognition.

A final aim of this study was to explore the presence of diametric effects of autistic and positive psychotic traits on social functioning. Contrary to recent exciting findings in non-clinical (Abu-Akel, Wood, Hansen, & Apperly, 2015, 2017b, 2017a, 2018a) and clinical (Abu-Akel et al., 2018b; Vaskinn & Abu-Akel, 2019) samples in the literature, our results do not provide further support for the diametric hypothesis. Although there are some differences in samples and methods across studies, the type, quality, and amount of data available to address this question in the GROUP cohort would have been sufficient to detect even small interaction effects if a diametric relation had been present. It is unclear how to interpret these opposing findings and we propose future studies address the diametric hypothesis by cross-validation in different samples with identical methods.

Several limitations should be considered when interpreting our findings. First, despite their psychometric strengths and common use, self-report questionnaires such as AQ-50 and SFS have notable shortcomings. For example, the AQ-50 is not equivalent to an objective assessment of autistic traits, potentially affected by subgroup bias (Agelink van Rentergem, Lever, & Geurts, 2019) and has a debatable factor structure (Lundqvist & Lindner, 2017). Additional observations, interviews, or informant reports are required to confirm the presence of traits and/or a clinical diagnosis of ASD in PD. Concurrently, it would help establish convergent validity of ASD assessments in PD and reduce potential bias introduced by mono-method associations (i.e. based on self-report). The SFS was constructed specifically to tap those areas of functioning that are crucial to individuals with schizophrenia and related disorders and generally has good psychometric properties, but its use may be suboptimal for assessing social functioning in individuals with ASD (Chan et al., 2019) and some domains may have low ecological validity (Schneider et al., 2017). Second, data were assessed approximately 6 years after baseline. Consequently, a relatively large portion of PD individuals (approximately 40%) fulfilled PANSS remission criteria. It is up to speculation if and how this affected the prevalence rates and relations with social functioning. Interestingly, when assessed *post hoc*, PD individuals in remission had significantly lower AQ scores than those not in remission, which could indicate that the impact of autistic traits extends beyond social functioning. Third, the data were cross-sectional and can therefore only establish concurrent relations and not whether autism traits are an actual determinant of poor outcome. Only follow-up data can and should address this in the future.

In conclusion, this report set out to establish the prevalence of (self-reported) autistic traits in an unprecedented sample size of individuals with PD and their unaffected siblings compared to typical comparisons. Our results confirm that the prevalence is overwhelmingly higher in PD, both categorically and dimensionally, an only marginally higher in FR on a dimensional level. The higher prevalence in PD is accompanied by a strong negative relation with social functioning on a behavioral level and to a lesser extent on a cognitive level. This indicates a need for better transdiagnostic clinical guidelines. For example, it is generally accepted that psychotic decompensation can occur due to a maladaptive stress response. For individuals on the autism spectrum, there are often clear indicators which situations trigger a severe stress response, e.g. sensory overload or unexpected, life-changing events. Therefore, when they present themselves with prodromal symptoms, these individuals can benefit from supervised daily schedules and access to a stimulus-free room to help prevent the onset/relapse of psychosis and potentially marginalize the need for antipsychotic treatment. These and other alternative treatment hypotheses need to be substantiated with additional scientific evidence (e.g. from clinical trials), but are worthy of consideration. Taken together, our findings suggest that autistic comorbidity in PD represents a very relevant clinical issue that needs to be addressed in order to improve treatment outcome.

## Supplementary material

For supplementary material accompanying this paper visit https://doi.org/10.1017/S0033291720000458. click here to view supplementary material
