# Supplementary material for: Autistic traits in psychotic disorders: prevalence, familial risk, and impact on social functioning
Source: Psychol Med. 2020 Mar 10;51(10):1704–13. doi: 10.1017/S0033291720000458 (PMC8327624; doi:10.1017/S0033291720000458)
Supplement: Supplementary file 1 [file S0033291720000458sup001.docx]

**Supplementary Tables**

S1. Spearman correlations of age, sex, IQ, trait and dependent variables for the total sample (above diagonal) and within the PD group (below diagonal)

|  | 1. | 2.^#^ | 3. | 4. | 5. | 6. | 7. | 8. | 9. |  |
| --- | --- | --- | --- | --- | --- | --- | --- | --- | --- | --- |
| 1. age |  | .026^**^ | -.011^**^ | -.078^**^ | -.050^**^ | -.057^**^ | .024^**^ | -.174^**^ | .081^**^ |  |
| 1. sex^#^ | -.010^**^ |  | -.021^**^ | -.158^**^ | -.064^**^ | .067^**^ | -.218^**^ | .015^**^ | .312^**^ |  |
| 1. IQ | -.043^**^ | -.063^**^ |  | -.225^**^ | -.105^**^ | -.096^**^ | -.254^**^ | .288^**^ | .301^**^ |  |
| 1. CAPE Positive | .024^**^ | -.007^**^ | -.128^**^ |  | .639^**^ | .601^**^ | .482^**^ | -.043^**^ | -.468^**^ |  |
| 1. CAPE Negative | .023^**^ | .090^**^ | .006^**^ | .561^**^ |  | .794^**^ | .488^**^ | .007^**^ | -.485^**^ |  |
| 1. CAPE Depressive | .001^**^ | .242^**^ | .041^**^ | .548^**^ | .746^**^ |  | .419^**^ | .012^**^ | -.393^**^ |  |
| 1. AQ Total | .090^**^ | -.032^**^ | -.172^**^ | .332^**^ | .358^**^ | .325^**^ |  | -.126^**^ | -.596^**^ |  |
| 1. PST-FB | -.155^**^ | -.019^**^ | .288^**^ | -.046^**^ | .026^**^ | .061^**^ | -.102^**^ |  | .105^**^ |  |
| 1. SFS | -.113^**^ | .179^**^ | .254^**^ | -.296^**^ | -.340^**^ | .238^**^ | -.496^**^ | .083^**^ |  |  |

^#^point-biserial correlations (male = 0; female =1); ^*^ *p* <.05; ^**^ *p* <.005

S2. *Generalized linear models for social functioning in PD group*

a. Dependent: Picture Sequencing Task – False Belief stories (x^2^ = 68.95, df = 6, p = .000)

|  | **B** | **SE** | **Waldχ^2^** | **df** | ***p*** |
| --- | --- | --- | --- | --- | --- |
| Total AQ | -.026 | .030 | .754 | 1 | .385 |
| CAPE Positive | -.009 | .023 | .159 | 1 | .690 |
| Age | -.145 | .031 | 22.301 | 1 | **< .001** |
| IQ | .070 | .011 | 44.549 | 1 | **< .001** |
| Total AQ * Age | -.007 | .004 | 2.989 | 1 | .084 |
| Total AQ * CAPE Positive | .000 | .003 | .012 | 1 | .963 |

b. Dependent: Social Functioning Scale – Total score (x^2^ = 230.89, df = 7, p = .000)

|  | **B** | **SE** | **Waldχ^2^** | **df** | ***p*** |
| --- | --- | --- | --- | --- | --- |
| Total AQ | -.475 | .402 | 86.494 | 1 | **<.001** |
| CAPE Positive | -.028 | .051 | .430 | 1 | .512 |
| CAPE Negative | -.267 | .043 | 23.793 | 1 | **< .001** |
| Age | -.102 | .055 | 4.559 | 1 | .033 |
| Sex | 4.026 | .048 | 38.687 | 1 | **< .001** |
| IQ | .089 | .011 | 23.574 | 1 | **< .001** |
| Total AQ * CAPE Positive | -.001 | .003 | .014 | 1 | .907 |
